# Role of Metastasis Suppressor KAI1/CD82 in Different Cancers

**DOI:** 10.1155/2021/9924473

**Published:** 2021-07-09

**Authors:** Wei Yan, Jinny Huang, Qian Zhang, Jian Zhang

**Affiliations:** ^1^Department of Surgery, Johns Hopkins University School of Medicine, Baltimore, MD, USA; ^2^Hepatobiliary Center of The First Affiliated Hospital, Nanjing Medical University and Research Unit of Liver Transplantation and Transplant Immunology, Chinese Academy of Medical Sciences, Nanjing, China; ^3^Department of General Surgery, The First Affiliated Hospital of Nanjing Medical University, Nanjing, China

## Abstract

Metastasis is one of the characteristics of malignant tumors and the main cause of death worldwide. The process of metastasis is mainly affected by tumor metastasis genes, tumor metastasis suppressor genes, tumor microenvironment, extracellular matrix degradation, and other factors. Thus, it is essential to elucidate the mechanism of metastasis and find the therapeutic targets in order to prevent the development of malignant tumors. KAI1/CD82, a member of tetraspanin superfamily of glycoproteins, has been reported as a tumor metastasis suppressor gene in various types of cancers without affecting the tumor formation. Many studies have demonstrated that low expression of KAI1/CD82 might lead to poor prognosis due to its interactions with other tetraspanins and integrins, resulting in the regulation of cell motility and invasion, cell-cell adhesion, and apoptosis. Considering its pathological and physiological significance, KAI1/CD82 could be a potential strategy for clinical predicting and preventing tumor progression and metastasis. The present review aims to discuss the role of KAI1/CD82 in metastasis for different cancers and examine its prospects as a metastasis biomarker and a therapeutic target.

## 1. Introduction

Metastasis has been the leading cause of cancer-related mortality. It is well known that tumor metastasis refers to the process in which malignant tumor cells leave the primary site, pass through vessels and then enter blood circulation and lymphatic circulation of the host, and eventually form malignant tumor, the same type of tumor as the primary one [1, 2]. Metastasis can be affected by a variety of molecular components [3]. Among them, tumor metastasis suppressor genes are a class of specific proteins which can negatively regulate tumor metastasis without affecting the growth of the primary tumor [4]. So far, over 20 metastasis suppressor genes have been discovered, and these genes are always found to be downregulated in different types of cancers [4, 5]. Therefore, targeting these suppressors is a promising therapeutic strategy for clinically inhibiting tumor metastasis.

Tetraspanins, a highly conserved protein family, are expressed on intracellular membranes and cell surface. 34 tetraspanins are known in mammals, of which 33 are expressed especially in humans [6]. Tetraspanins could affect multiple cell processes including motility, antigen presentation, and receptor-mediated signaling [7]. KAI1, also known as CD82, consists of four transmembrane domains and is a metastasis suppressor gene belonging to the tetraspanin superfamily located on chromosome 11p11.2 [8, 9].

A large number of experiments have shown that the differentially expressed KAI1/CD82 is closely related to malignant tumors, which can be served as a biomarker in tumors [10]. KAI1/CD82 gene was initially cloned as a human gene that could inhibit the metastatic ability of rat AT6.1 prostate cancer cells. In a mouse xenograft model, it was shown that enforced expression of KAI1/CD82 could repress lung metastasis of AT6.1 cells without affecting the growth rate of the primary tumor [11]. KAI1/CD82 expression is decreased in malignant tumors and is closely related to malignant progression, metastasis, and prognosis, containing breast, colon, lung, ovarian, nasopharyngeal, liver, and pancreatic cancer [12–14]. In addition, KAI1/CD82 influences diverse biologic functions, including cell signal transduction, adhesion, migration, motility, protein trafficking, and aggregation [9]. A recent study showed that using peptide mimicking the small extracellular ring domain of KAI1/CD82 could suppress cell metastasis, invasion, and adherence in vitro and inhibit the pulmonary metastasis in vivo [15].

This review will mainly focus on the understanding of the behavior of KAI1/CD82 in different types of cancers and discuss the mechanism and pathways through which it plays a role in tumor metastasis.

## 2. Different Cancers

### 2.1. Hepatocellular Carcinoma

Despite the clinically therapeutic progress of hepatocellular carcinoma (HCC) such as liver surgical resection, transplantation, or chemotherapy, the survival rate was less than 5% within five years, mainly due to the high rate of postoperative recurrence and metastasis after surgical resection [16, 17].

Firstly, it is well-known that the KAI1/CD82 mRNA and protein expression was significantly reduced in the tumors compared to adjacent nontumor liver tissues obtained from the same patient [18]. Previous research reported that low expression of miR-362-3p can cause high expression of KAI1/CD82, which suppresses migration and invasion of HCC cells [19]. Additionally, except for miR-363-3p, KAI1/CD82 was also confirmed to be downregulated by miR-197, while anti-miR-197 could suppress HCC migration and invasion by targeting KAI1/CD82 [18].

Moreover, a study found that the synergistic effects of KAI1/CD82 and ganglioside GM3 or GM2/GM3 were able to inhibit HCC cell motility and migration via epidermal growth factor receptor (EGFR) or cMet-activated Pl3K/Akt signaling pathway [20]. In addition, p53 dysfunction and low expression of JunB (a member of the fos/jun family) are simultaneous, while they may play an important role in downregulating the expression of KAI1/CD82 in HCC [21]. Furthermore, KAI1/CD82 was also demonstrated to suppress HGF-induced migration of hepatoma cells via upregulation of Sprouty2 [22]. A latest study also proposed that HBV may inhibit the expression of KAI1/CD82 through hypermethylation of the promoter in hepatoma cells, leading to the development of HCC [23].

### 2.2. Gastric Cancer

Gastric cancer (GC) is one of the most common tumors and the third leading cause of cancer death worldwide [24]. Although great improvements and progress such as surgery and adjuvant chemotherapy have been made, the overall five-year survival rate of GC still remains low, largely due to the lack of typical early symptoms, which means most patients have developed advanced-stage tumor at diagnosis, accompanied with distant metastasis [25]. However, little exploration has been made on promising biomarkers that could decrease the risk of recurrence and metastasis [26].

At both the mRNA and protein level, poorly differentiated GC cell lines showed higher expression of miR-362-3p and lower expression of KAI1/CD82 compared to highly differentiated GC cell lines [19]. Downregulation of miR-362-3p and upregulation of KAI1/CD82, which may mediate E-cadherin, N-cadherin, and vimentin expression in GC cells, could result in the inhibition of GC migration and invasion [27].

Furthermore, KAI1/CD82, which could also be regulated by miR-197, suppresses EGFR expression and phosphorylation in EGF- and HGF-dependent manner in Hepa1-6 cells. KAI1/CD82 knockout significantly promoted the invasion of GC cells through activating the EGFR/ERK1/2-MMP7 pathway [28]. These findings all indicated that KAI1/CD82 may be involved in the process of GC occurrence and development.

### 2.3. Esophageal Squamous Cell Carcinoma

Esophageal squamous cell carcinoma (ESCC) has the seventh highest incidence and the sixth highest cancer-related mortality worldwide [29]. Because the progression of ESCC is characterized by natural aggression and asymptomatic behavior, the diagnosis of ESCC is usually at an advanced stage, with tumor cells invading the adventitia and metastatic cells entering local and distant lymph nodes [30].

Studies found lowly expressed KAI1/CD82 was significantly correlated with the pathological stage, lymph node metastasis, and poor prognosis in ESCC patients. In ESCC cells, overexpression of KAI1/CD82 significantly decreased TGF-*β*1/Smad signal pathway, including TGF-*β*1, Smad2/3, MMP-2, and MMP-9 level, and thus decreased cell proliferation, invasion, and metastasis [31].

Besides, motility-related protein (MRP-1/CD9), which is considered to inhibit cell motility, could be inversely correlated with lymph node metastasis in ESCC through downregulating KAI1/CD82 expression [32].

### 2.4. Colorectal Carcinoma

Colorectal carcinoma (CRC) consists of the development of cancer from colon or rectum [33]. CRC ranks third among malignancies worldwide and is the second leading cause of cancer-related deaths [34]. The clinical treatment of CRC mainly depends on the stage of the disease progression, molecular analysis, and the patients' health status [35].

Decreased KAI1/CD82 expression is correlated with distant metastasis and TNM stage, while enhanced KAI1/CD82 expression is also positively correlated with overall survival [36, 37].

Additionally, a previous study also proposed that the KAI1/CD82 expression did not alter in vitro colorectal carcinoma cell proliferation, but the apparent increased ability of cell-cell adhesion and cell aggregation was observed in KAI1/CD82 transfected cells [38]. 90K, a tumor-associated glycoprotein, which could interact with KAI1/CD82, showed antitumor activity in colorectal cancer cells via suppressing Wnt signaling with a novel mechanism of *β*-catenin ubiquitination [39].

### 2.5. Breast Cancer

Breast cancer (BC) is the most frequently diagnosed cancer in women. It has a variety of tumor heterogeneity, which poses a challenge to the traditional biomarkers for early detection and prognosis [40]. The diagnosis of breast cancer mainly depends on imaging techniques and histopathological examination, but such diagnosis has obvious defects of hysteresis and invasion.

KAI1/CD82 expression was apparently lower in tumors than in adjacent tissues and benign breast disease tissues. Lacking KAI1 expression might be closely associated with more aggressive form of breast cancer [41]. Besides, KAI1/CD82 expression in tissues shaded negatively with the level of KAI1/CD82 in exosomes, indicating that the KAI1/CD82 expression was redistributed from tissues to the blood as breast cancer developed and metastasized [42]. Additionally, knockdown of liprin-*α*1 protein in breast cancer cells MDA-MB-231 and Hs578T controls cell edge protrusions during invasion and metastasis and is encoded by PPF1A1, which could lead to the upregulation of KAI1/CD82 [43]. Furthermore, another study identified and characterized a primary nuclear nonpolyadenylated antisense- (as-) lncRNA, named lncRNA SKAI1BC and representing as “suppressor of KAI1 in breast cancer”, could suppress KAI1/CD82 mRNA and protein expression in triple-negative breast cancer cell line MDA-MB-231 [44].

### 2.6. Lung Cancer

Nowadays, with continuous environment and lifestyle changes, the incidence of lung cancer (LC) has been increasing. It is now the most common cancer worldwide. Despite increasing knowledge in the development and risks of lung cancer, it remains the leading cause of death in both men and women worldwide. Approximately 85% of lung cancer patients are non-small cell lung cancer (NSCLC) [45].

The study found that the KAI1/CD82 level was significantly lower in lung tissues of NSCLC patients compared to normal lung tissues. KAI1/CD82 level was negatively correlated with tumor size, metastasis, and TNM stage in NSCLC [46, 47]. There are several pathways by which KAI1/CD82 could suppress lung cancer metastasis. First, KAI1/CD82 enhances the expression of miR-203 and downregulates FZD2 expression, thus suppressing metastasis by inhibiting the Wnt signaling pathway in NSCLC cells [45]. Second, KAI1/CD82 can also inhibit the Wnt signaling pathway by weakening EGFR signaling, which may attenuate metastatic colonization [48]. Downregulation of KAI1/CD82 could be a critical step involved in EGFR overexpression, and EGFR mutations trigger stronger tumorigenic activity [49]. Third, the invasion, progression, and metastasis of NSCLC cells were shown to be positively correlated with E-CAD and related to low CD82 expression. CD82 may stabilize or strengthen E-cad-dependent intercellular adhesion by regulating *β*-catenin-mediated signal transduction on NSCLC cells and prevent cancer cells from seceding from the primary tumor site [50]. Fourth, KAI1/CD82 downregulated the Rac1 expression through the PI3K/Akt/mTOR pathway and reduced the metastatic phenotype of H1299 cells [51]. Lastly, KAI1/CD82 overexpression in the H1299 cells suppresses the tumor invasiveness and metastatic potential of NSCLC cells by inducing MMP9 inactivation via the upregulation of TIMP1 [52].

### 2.7. Nasopharyngeal Carcinoma

Nasopharyngeal carcinoma (NPC) is an epithelial carcinoma arising from the nasopharyngeal mucosal lining, with significant geographical differences [53]. NPC generally remains undetected, and diagnosis is often delayed until an advanced stage, when distal-organ metastasis is common [54].

Studies found that the KAI1/CD82 gene was highly expressed in low metastatic NPC cell lines and nonneoplastic NPC tissues, while lowly expressed in high metastatic potential NPC cells and NPC tissues [55]. It was demonstrated that compared with the control group, tumor growth was not suppressed when mice were injected with KAI1/CD82^+^ EPCs (endothelial progenitor cells), but the incidence of lung metastasis and the number of metastatic foci on the lung surface were significantly reduced [14].

### 2.8. Prostate Cancer

Prostate cancer (PC) is the most frequently diagnosed cancer in 105 countries in men and is the most common cause of death from malignant tumours [56]. Most PCs are curable, but metastatic forms are associated with lower survival rate; therefore, imaging is needed to detect and track metastases evolution during treatment [57].

In a decade-long free survival study of biochemical failure, men who used KAI1/CD82 negative CPCs treated by radical prostatectomy had a higher rate of biochemical failure compared with men who used positive PCPs [58]. A present study suggests that KAI1/CD82 functions in suppressing TGF‐*β*1‐ and Wnt‐induced EMT in prostate cancer cells by inhibiting the TGF‐*β*1/Smad and Wnt/*β*-catenin pathways, resulting in the development of a motile and invasive mesenchymal phenotype related to the initiation of the metastatic cascade [11]. Another result pointed out that KAI1/CD82 suppressed adhesion signaling pathways through lateral interactions with *α*_3_*β*_1_ and *α*_5_*β*_1_ integrin, thereby inhibiting EMT adhesion of prostate cancer cells to fibronectin matrix and reducing cell migration and invasion ability [59]. In addition, KAI1/CD82 attenuated the activation of *β*1 integrin and downregulated its outside-in signaling. This results in the reduction of focal adhesion formation and fibronectin expression/secretion, which subsequently interferes with cell adhesion properties and motility in human prostate cancer cells [60]. Furthermore, EWI2/PGRL, an immunoglobulin superfamily member, could synergize KAI1/CD82 to inhibit the migration of prostate cancer cells [61, 62].

### 2.9. Melanoma

Melanoma is a type of skin cancer that originates from melanocytes, the specialized melanin-producing cells that mainly reside in the skin [63]. The incidence and mortality rates of melanoma cancer are increasing due to its great potential to metastasize, and it is the leading cause of skin cancer-related deaths [64].

Studies found that downregulation of KAI1/CD82 mRNA expression in human melanoma cell lines was related to loss of heterozygosity or allelic imbalance [65]. One recently discovered function of KAI1/CD82 is to decrease tumor aggressiveness by suppressing U2AF2-mediated CD44 alternative splicing. Overexpression of CD82 suppressed U2AF2 activity by inducing U2AF2 ubiquitination [66]. This may have potential prognostic and therapeutic implications in melanoma.

KAI1/CD82 mRNA was also identified as a direct target mRNA of miR-338-5p, which is correlated with tumor stage, metastasis, and survival rate [67]. KAI1/CD82 can suppress the ability of melanoma cell invasion and migration by reducing the activity of metalloproteinase-2 or by using another tumor suppressor protein called inhibitor of growth 4, which regulates p65 [68]. In highly metastatic melanoma cells, the expression of KAI1/CD82 increased p21 expression upon binding of the Duffy antigen receptor group (DARC). This induces tumor cell senescence and interrupts IL-8-mediated vascular endothelial- (VE-) cadherin disassembly [66]. KAI1/CD82 expression also inhibited melanoma cell-induced gap formation, melanoma cell extravasation in vitro, and subsequent lung metastasis development in vivo [69].

### 2.10. Acute Myelogenous Leukemia

Acute myelogenous leukemia (AML) originates from hematopoietic stem/progenitor cells and is characterized by the increased numbers of immature myeloid blasts in the bone marrow (BM). AML accounts for approximately 20% of acute leukemia in children and adolescents [70].

Studies show that KAI1/CD82 inhibits matrix metalloproteinase 9 and augments adhesion of CD34^+^/CD38^−^ AML cells to the BM microenvironment [71, 72]. KAI1/CD82 can also positively regulate the expression and phosphorylation of EZH2 via inactivation of p38 MAPK signaling in leukemia cells, thus suppressing differentiation programs in leukemic stem cells and augmenting their leukemogenic activity [73]. A recent study revealed a positive correlation between CD82 and BCL2L12 expression (an antiapoptotic protein) at mRNA and protein levels due to STAT5A and AKT signaling in AML cells isolated from patients, which eventually stimulated proliferation and engrafting of leukemia cells [74]. Furthermore, CD82 positively regulated the STAT5/IL‐10 signaling pathway in CD34^+^/CD38^−^ AML cells. Downregulation of CD82 dephosphorylated STAT5 which could bind to the promoter region of the IL‐10 gene and stimulated IL‐10 expression at the transcriptional level, resulting in the decreased level of IL‐10 in CD34^+^/CD38^−^ AML cells [75]. In addition, in AML children, KAI1/CD82 mRNA expression increases the expression of downstream molecules in the Wnt/*β*-catenin pathway such as *β*-catenin, c-myc, cyclinD1, and survivin. These molecules will regulate the proliferation and chemotherapy resistance of AML cells [76]. Another study also shows that an aggressive leukemia phenotype in AML children is related to a mechanism where KAI1/CD82 membrane organization regulates sustained PKC*α* signaling [77].

KAI1/CD82 may also serve an important role in the evolution of pediatric acute lymphoblastic leukemia (ALL). The KAI1/CD82 mRNA expression level was significantly higher in the patients with ALL-ND (newly diagnosed), B-cell-ALL, and T-cell-ALL compared with those aged match children without BM disease [78].

## 3. Conclusion

**Figure 1:**
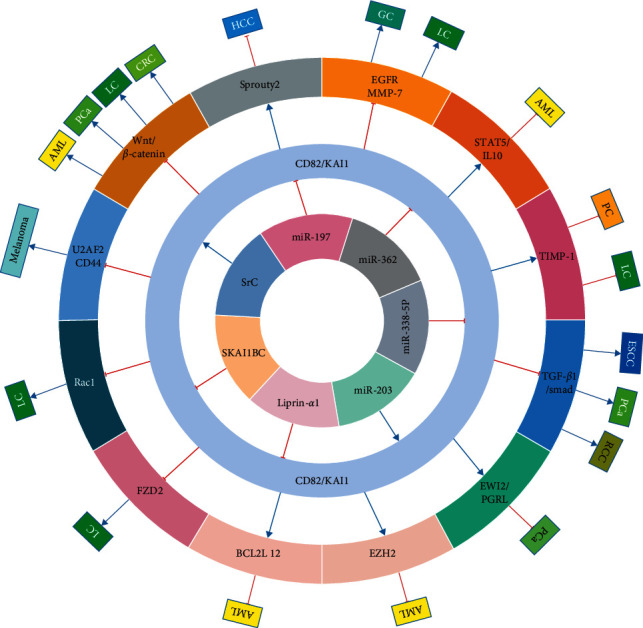
Summary of current research on KAI1/CD82 in diverse cancers. HCC: hepatocellular carcinoma; GC: gastric cancer; ESCC: esophageal squamous cell carcinoma; CRC: colorectal carcinoma; BC: breast cancer; LC: lung cancer; NPC: nasopharyngeal carcinoma; PC: prostate cancer; RCC: renal cell carcinoma; AML: acute myelogenous leukemia.

KAI1/CD82 is a well-characterized metastasis suppressor of various solid malignant tumors without affecting primary tumor growth and a recognized biomarker to predict metastatic potential (Figure 1). In detail, KAI1/CD82 deficiency is related to aggressive tumor behaviors, such as high drug resistance, low differentiation grade, and high recurrence rate, as well as decreased disease free and overall survival duration. According to the research above, KAI1/CD82 is an independent prognostic factor that can predict survival for various tumors, suggesting that it can be used as a novel diagnostic and prognostic biomarker.

Although the structure and function of KAI1/CD82 are well understood, no agent targeting KAI1/CD82 expression or its function is currently available to be used in clinical metastasis therapy. Due to its structure as a transmembrane protein, the standard methods for drug delivery cannot be applied; however, several other strategies can be taken into consideration. Endogenous locus could be induced to upregulate the expression of KAI1/CD82 and viral-mediated gene therapy methods may be used to restore its function. Other potential targets for cancer metastasis therapy could include specifically expressed downstream pathways which are regulated by the loss of KAI1/CD82 or some extracellular domains of KAI1/CD82 which may show tumor metastasis function. Future studies are still needed to fully explicate the role of KAI1/CD82 in certain cancers and also address whether KAI1/CD82 downregulation is a cause or consequence of the progression of cancer. In summary, KAI1/CD82 may be considered a significant prognostic marker in predicting metastatic manifestation and may be a promising and effective candidate for treatment for certain cancers.
